# High-Performance Waveguide-Integrated Ge/Si Avalanche Photodetector with Lateral Multiplication Region

**DOI:** 10.3390/mi13050649

**Published:** 2022-04-19

**Authors:** Daoqun Liu, Peng Zhang, Bo Tang, Wenwu Wang, Zhihua Li

**Affiliations:** 1 Institute of Microelectronics, Chinese Academy of Sciences, Beijing 100029, China; liudaoqun@ime.ac.cn (D.L.); zhangpeng1@ime.ac.cn (P.Z.); tangbo@ime.ac.cn (B.T.); wangwenwu@ime.ac.cn (W.W.); 2 School of Electronic Electrical and Communication Engineering, University of Chinese Academy of Sciences, Beijing 100049, China

**Keywords:** photodetector, avalanche multiplication, waveguide integration, Ge/Si heterojunction

## Abstract

High-performance waveguide-integrated Ge/Si APDs in separate absorption, charge, and multiplication (SACM) schemes have been exploited to facilitate energy-efficient optical communication and interconnects. However, the charge layer design is complex and time-consuming. A waveguide-integrated Ge/Si avalanche photodetector (APD) is proposed in a separate absorption and multiplication (SAM) configuration. The device can work at low voltage and high speed with a lateral multiplication region without complexity of the charge layer. The proposed device is implemented by the complementary metal-oxide-semiconductor (CMOS) process in the 8-inch Si photonics platform. The device has a low breakdown voltage of 12 V and shows high responsivity of 15.1 A/W at 1550 nm wavelength under optical power of −22.49 dBm, corresponding to a multiplication gain of 18.1. Moreover, an opto-electrical bandwidth of 20.7 GHz is measured at 10.6 V. The high-speed performance at low voltage shows a great potential to implement high-energy-efficient Si optical communications and interconnections.

## 1. Introduction

Near-infrared photodetectors have been widely used in fiber communication [1,2,3,4,5], spectroscopy [6,7], and light detection and ranging (LIDAR) [8,9]. High-speed and high-sensitivity photodetectors are desirable to relax the power budget and improve the energy efficiency of fiber communication. Thus, avalanche photodetectors (APDs) with internal multiplication gain are preferable because of higher sensitivity than p-i-n photodetectors [10]. In terms of material, photodetectors based on III-V compound semiconductors have been widely applied for the weak light detection for fiber communication due to their low dark current and high speed. Nevertheless, high costs and the inability of monolithic integration with microelectronic devices have hindered the further promotion of III-V photodetectors. Furthermore, III-V APDs generally are noisy due to the high k-value (the ratio of ionization coefficient of the hole to that of the electron) of III-V semiconductors. Fortunately, photodetector based on Ge/Si heterojunction is a promising alternative due to their compatibility with CMOS and the excellent avalanche multiplication performance of silicon.

In recent years, high-performance waveguide-integrated Ge/Si APDs have been intensively explored to improve the gain-bandwidth product (GPB) and sensitivity [11]. The Ge-only APDs leveraging lateral [12,13] or vertical p-i-n [5,14,15] configurations can achieve high bandwidth at low voltage. But these APDs suffer from the high dark current originating from too strong electric field inside the germanium. The thin charge layer in the SACM Ge/Si APD [9,16,17,18,19] could suppress the dark current by controlling the electric field inside the Ge layer. Generally, the charge layer (~0.1 μm) is formed by implanting a thick (>0.6 μm) epitaxial Si layer with a p-type dopant. However, the thick epi-Si would deteriorate light coupling from the Si waveguide into the Ge region. Furthermore, the thick Si epitaxy is incompatible with the modern CMOS process and leads to higher operating voltage (>20 V). Although SACM Ge/Si APDs with the lateral multiplication region and the charge region [2,20,21,22,23] eliminate the requirement of Si epitaxy, designing doping profile inside the charge layer is complex. Recently, Ge/Si APD in three-terminals layout was demonstrated [24], which can achieve low voltage operation by independently controlling electric field in Ge region and multiplication region. However, the Si epitaxy is still needed, and the metal contacts on the germanium layer is harmful to the responsivity. Therefore, it is desirable to achieve comparable performance with a simpler structure, and consequently a lower cost.

In this work, a waveguide-integrated SAM Ge/Si APD with the lateral multiplication region is designed. The proposed device can obtain high bandwidth at low voltage without the complex charge layer and the contacts on germanium. The device was fabricated through the CMOS processes. The static characteristics, including dark current and multiplication gain, were measured and analyzed in detail. The extracted breakdown voltage is as low as 12 V, and the high responsivity of 15.1 A/W under optical power of −22.49 dBm can be extracted at 1550 nm wavelength. The 20.7 GHz bandwidth is measured at −10.6 V through a small-signal measurement.

## 2. Design and Fabrication

**Figure 1:**
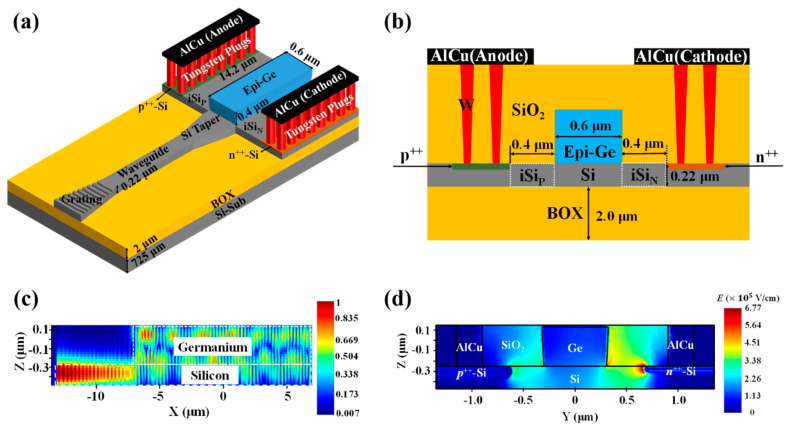
Structure of the waveguide-integrated SAM Ge/Si APD. (**a**) 3D schematic of the device; (**b**) the cross-section of the device; (**c**) the electric component of the optical field inside the device (simulation using Lumerical FDTD); (**d**) the electric field profile inside the device at avalanche breakdown (simulation using Lumerical DEVICE).

Figure 1a shows the 3-D schematic of the fabricated waveguide-integrated SAM Ge/Si APD. Light couples from an optical fiber into the single-mode Si waveguide through the focusing surface grating coupler. The Si taper at the end of the waveguide can reduce the mode mismatch between the waveguide and the multimode region. This multimode region consists of the top Si layer and the Ge region. The optical mode oscillates between the Si and Ge in the multimode region until fully absorbed by the Ge region, as shown in Figure 1c. Photogenerated holes inside the Ge region can be collected by the anode through an intrinsic Si region adjacent to the p^++^-Si region (labeled as iSi_P_). On the other hand, photogenerated electrons are injected from the Ge region into another intrinsic Si region (labeled as iSi_N_) at which the high electric field is confined, as shown in Figure 1d. Compared to conventional vertical design [4,14,15,21,24], there is no energy dissipation introduced by light absorbing from the contact metal on the germanium. For the SACM APD, the charge layer can restrict the electric field inside the Ge region and multiplication region to a reasonable level. On the contrary, for the proposed structure in this paper, such restriction is achieved by the dielectric constant difference between Si and Ge as well as the background doping inside the Ge layer [25].

**Figure 2:**
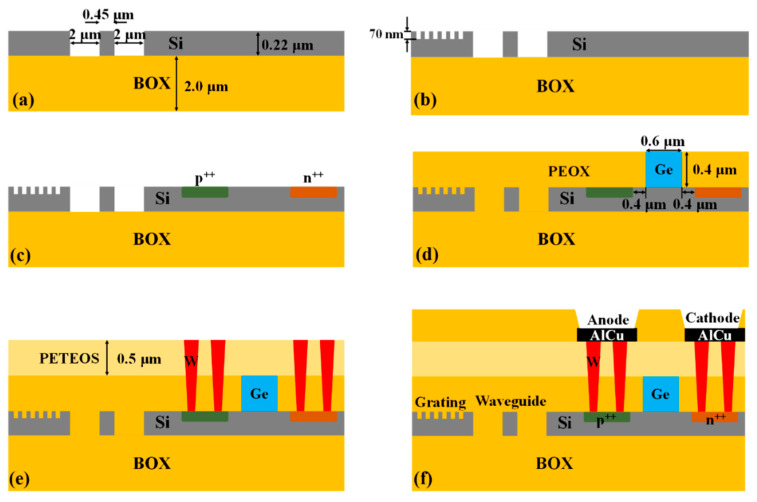
The key processes for waveguide-integrated SAM Ge/Si APD. (**a**) Fabrication of the channel waveguide; (**b**) Fabrication of the grating; (**c**) P-type and N-type implantation for Ohm-contact; (**d**) Selective epitaxial growth of germanium; (**e**) Formation of tungsten plugs; (**f**) Metal deposition, patterning and device passivation.

**Figure 3:**
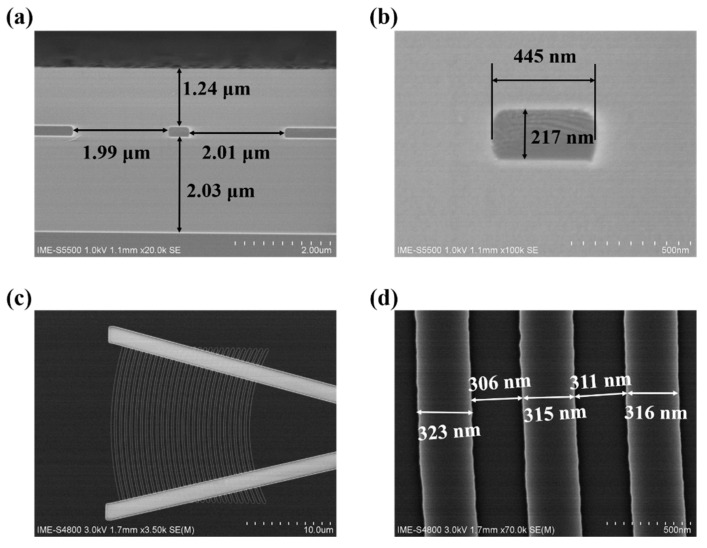
SEM image of waveguide and gratings. (**a**) Cross-section SEM image of the optical waveguide; (**b**) Cross-section SEM image of the core of the waveguide; (**c**) Topography SEM image of the grating (overview); (**d**) Topography SEM image of the grating (Zoom-in view).

**Figure 4:**
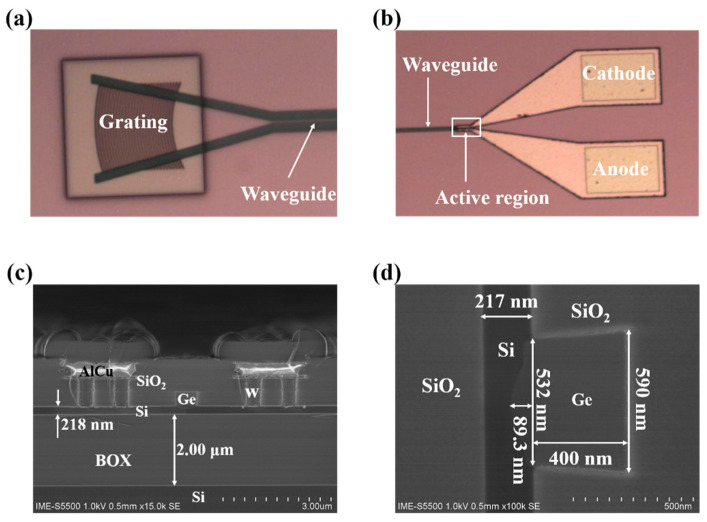
(**a**) Optical microscopy image of the grating; (**b**) Optical microscopy image of the active area of the device; (**c**) Cross-section SEM image of the active region; (**d**) Cross-section of the germanium region.

The waveguide-integrated SAM Ge/Si APD was fabricated on an 8-inch SOI wafer with a 220 nm-thick p-type <100>-oriented top Si layer and a 2 μm-thick buried oxide (BOX) layer. Figure 2 shows the critical process to fabricate the device. Firstly, the channel waveguide was fabricated by a 220 nm-depth dry etching, as shown in Figure 2a and Figure 3a. The nominal width of the channel waveguide is designed to be 450 nm to support the single-mode. The measured width of the fabricated waveguide is 445 nm, as shown in Figure 3b. The curved sidewall of the waveguide was caused by high-temperature annealing under hydrogen ambient, which has been developed to reduce the scattering loss [26,27]. Secondly, the grating was formed by a 70 nm-depth dry etching process, as shown in Figure 2b. The designed grating has a period of 630 nm and duty of 0.5 to coupling 1550 nm-light from the fiber to the waveguide. The fabricated grating is shown in Figure 3c and has the period of 628 nm (mean value) and the duty of 0.51 (mean value), as shown in Figure 3d. Thirdly, specific regions on the top silicon layer were sequentially implanted with boron and phosphorus ions, as shown in Figure 2c, followed by the rapid thermal annealing to activate those ions and repair the implantation damage. Fourthly, a 1 μm-thick oxide was deposited on the wafer by plasma-enhanced chemical vapor deposition (PECVD) technology. Then, the chemical mechanical polish (CMP) process was used to planarize the wafer then the thickness of the oxide was reduced to 500 nm. After etching out a 14.2 μm × 0.6 μm × 0.5 μm (length × width × depth) window and careful cleaning, germanium epitaxial growth was conducted by the reduced pressure chemical vapor deposition (RPCVD) technology. Another CMP step was used to remove the overgrown part of the germanium and to planarize the wafer. It is worth noting that the germanium is always over-polished during the CMP due to the absence of in-line thickness monitoring. Thus, the remaining Ge is about 400 nm, as shown in Figure 2d and Figure 4d. The metallization and passivation were accomplished by the standardized back-end-of-line (BEOL) process, as shown in Figure 2e,f. The finished device is shown in Figure 4a,b show the optical microscopy image of the grating and active area of the device, respectively. Figure 4c,d show the cross-section of the active region and the germanium region, respectively.

## 3. Device Characterization

### 3.1. Waveguide and Grating Loss

**Figure 5:**
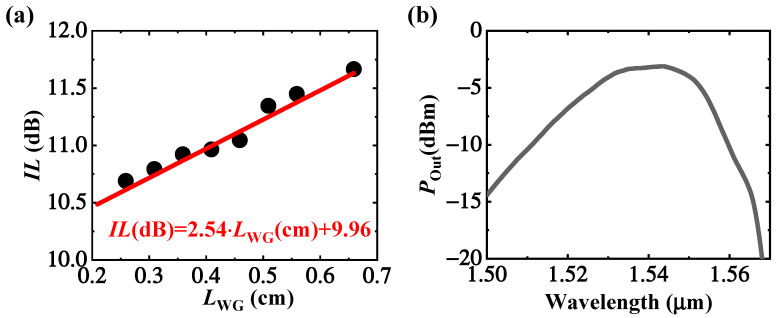
(**a**) Insertion loss of the “grating-waveguide-grating” structure is the function of the waveguide length (the black dot is measured data and the solid red line is the linear fitted result); (**b**) The optical response spectrum of the grating.

Generally, the grating coupling loss and waveguide propagation loss should be characterized before the active-device testing. The grating and waveguide loss is extracted by testing the insertion loss of “grating-waveguide-grating” structures with different waveguide lengths under 1550 nm, as shown in Figure 5a. The red solid linear-fitted line slope is about 2.54 dB/cm and equals the propagation loss of the waveguide. Half of the linear fitted line intercept with the vertical axis is about 4.98 dB and equals the grating coupling loss. The optical spectrum response of the grating is shown in Figure 5b. It shows the peak wavelength of 1543 nm, and the tiny shift from the designed value of 1550 nm may be attributed to the process variation as depicted in Figure 3d.

### 3.2. Static Photoresponse

**Figure 6:**
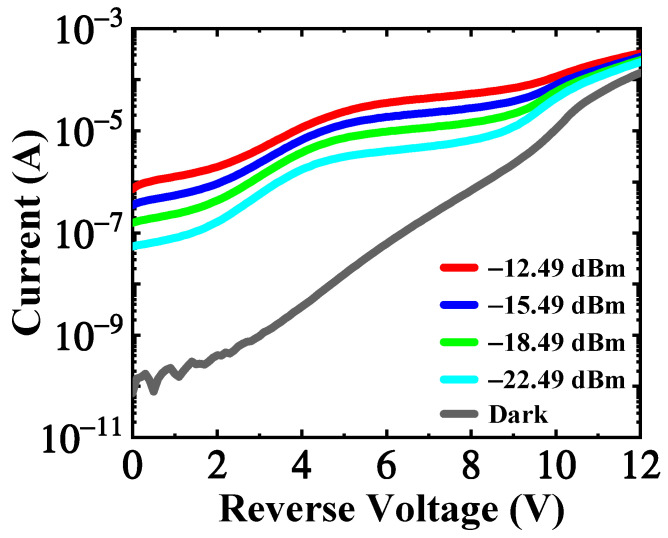
The dark current and photocurrent under different incident optical power are the function of reverse voltage.

The dark current measurement was performed without light illuminated at room temperature. The dark current versus reverse voltage characteristics is shown in Figure 6. The dark current starts at a very low value of 74 pA at 0 V and goes up to 135 μA around 12 V which is defined as the device’s breakdown voltage. The measurement system leakage current and probe capacitance contribute to the oscillation in the dark current at low bias from 0 V to 3 V. The dark current increases with reverse voltage in a near-exponential way from 3 V to 10 V, which relates to the gradually depleting the germanium layer and underlying Si layer.

At the wavelength of 1.55 μm, the photo-response of the APD was characterized under different input optical powers. The photocurrent versus voltage characteristics is also shown in Figure 6. The increasing trend of the photocurrent is similar to that of the Al_0.8_In_0.2_As_0.23_Sb_0.77_ APD developed by A.K. Rockwell, et al. [28] The slow rise of photocurrent from 0 V to 5 V might also result from the heterojunction barrier. However, the increase in photocurrent from 5 V to 9 V results from the improved carrier collection efficiency and avalanche multiplication. At the reverse voltage of 9 V, the germanium region should be completely depleted. Therefore, beyond 9 V, the increase in photocurrent can be attributed to the enhanced avalanche multiplication. The band structure under different voltages, as shown in Figure S3 in the Supplementary Material, can help to visulize above analsysis. More detail analaysis about the carrier transportation mechanism at different voltages can be found in the Section A of the Supplementary.

**Figure 7:**
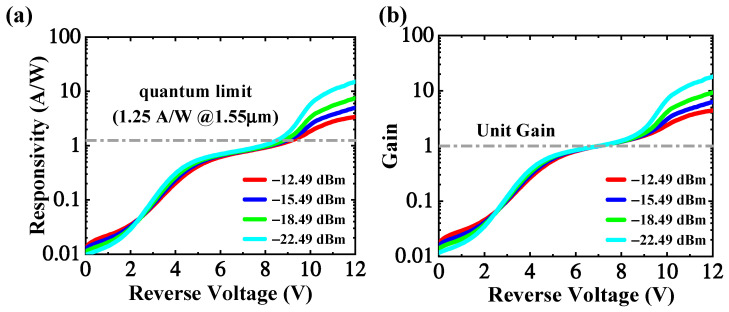
(**a**) The extracted responsivity is the function of the reverse voltage (the inset is the responsivity at low voltage); (**b**) The extracted multiplication gain is the function of the reverse voltage.

The responsivity is defined as the ratio of net photocurrent to the incident optical power, and can be calculated according to the following formula:

(1) $$R\left(V\right)=\frac{I_{net}\left(V\right)}{P_{in}}=\frac{I_{ph}\left(V\right)-I_{d}\left(V\right)}{P_{in}}$$

where *I_net_*, *I_ph_*, and *I_d_* are the net photocurrent, total photocurrent, and dark current, respectively. The *P_in_* is the optical power injected into the Ge/Si multimode region from the Si taper. Although the grating and the waveguide loss were kicked out when estimating the *P*_in_, the coupling loss from the waveguide to the Ge layer is hard to estimate. Therefore, the responsivity may be underestimated. The extracted responsivity versus reverse voltage characteristics is depicted in Figure 7a. The poor responsivity under low bias (0~5 V) may be attributed to the following factors. The weak electric field inside the germanium region hampers the separation of photogenerated electron-hole pairs. Besides, the GeSi alloy, induced by Ge and Si atom interdiffusion during epitaxy, has a much lower light absorption coefficient and consequently deteriorates responsivity [29,30]. Under the reverse voltage of 8 V and −22.49 dBm optical power, the responsivity reaches the quantum limit of 1.25 A/W, corresponding to the unit external quantum efficiency. Generally, the external quantum efficiency of a p-i-n photodetector hardly reaches 100%. Therefore, avalanche multiplication should be already triggered below 8 V. However, it is difficult to determine the exact value of the reverse voltage at which the avalanche multiplication emerges.

The multiplication gain is defined as the ratio of the net multiplied photocurrent to the primary one, and can be evaluated through the following formula:

(2) $$G\left(V\right)=\frac{I_{ph}\left(V\right)-I_{d}\left(V\right)}{I_{ph}\left(V_{ref}\right)-I_{d}\left(V_{ref}\right)}=\frac{R\left(V\right)}{R_{0}}$$

where *V_ref_* is the unit gain voltage, *R*_0_ is the primary responsivity. The unit gain voltage is selected to be 7.0 V in this work. The primary responsivity of the fabricated device has a mean value of 0.80 A/W. The tiny discrepancy among responsivity/gain versus voltage characteristics below the unit gain voltage may be caused by the system error. The gain reaches the maximum value of 4.4, 6.3, 9.3, and 18.1 at the breakdown point under optical power of −12.49 dBm, −15.49 dBm, −18.49 dBm, and −22.49 dBm, respectively. There is a notable trend that, at the same high voltage (>8 V), the responsivity and gain rise with the decrease in optical power. The space charge effect [31] may contribute to such a trend, which can be verified by the electric field profile inside the multiplication region, as shown in Figure S4 in the Section B of the Supplementary Material.

### 3.3. Small-Signal Characteristics

**Figure 8:**
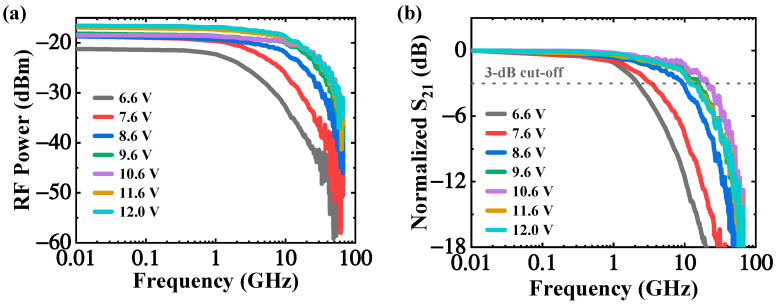
(**a**) The measured RF power under different voltages; (**b**) the normalized S21 parameter at different voltages.

**Figure 9:**
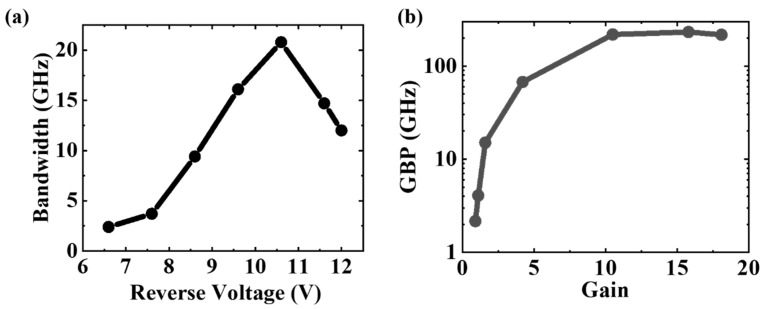
(**a**) The extracted 3-dB bandwidth is the function of the reverse voltage; (**b**) the extracted gain-bandwidth product is the function of the reverse voltage.

The frequency response of the APD is a key figure of merit for high-speed applications. In this work, the frequency response performance of the APD was evaluated by the small-signal radio-frequency (RF) measurement using a light-wave component analyzer. The calibration for the RF testing system was performed to eliminate the parasitic effects of instruments on the device. At 1550 nm wavelength and −22.49 dBm, the measured RF response at different voltages is shown in Figure 8. As the presence of avalanche multiplication, the low-frequency RF power increases with reverse voltage [22], as shown in Figure 8a. The normalized S21 parameter versus RF frequency was plotted in the Figure 8b. The APD shows a low 3-dB bandwidth of 2.2 GH at −6.6 V because of the weak electric field inside the Ge region. As shown in Figure 9a, the 3 dB bandwidth increases with reverse bias and reaches the maximum of 20.7 GHz at −10.6 V, indicating that the bandwidth is mainly limited by the photogenerated carrier transit time [22]. However, beyond −10.6 V, the avalanche build-up time dominates the APD frequency response and causes a drastic decline in 3-dB bandwidth [13]. More details about the relationship between the bandwidth and reverse voltages can be found in the Section C of the Supplementary Material.

The gain-bandwidth product (GBP) is a crucial indicator of avalanche photodiode for high-speed digital communication applications. As depicted in Figure 9b, the GBP increases with reverse voltage and reaches a plateau around 10.6 V. A maximum gain-bandwidth product of 232 GHz is extracted at 11.6 V.

## 4. Benchmarking and Outlook

Table I benchmarks the structure and process complexity and the overall performance of the proposed device in this work by comparing the key parameters with other state-of-the-art waveguide-integrated Ge/Si APD. In Table 1, the column titled “IMP Layers” indicates the number of layers that should be allocated to ion implantation when designing the avalanche photodiode layout. The column titled “Epi-Si” shows whether there is a requirement for silicon epitaxy or not. The column titled “Recess-Ge” indicates whether the germanium layer is selectively grown on a silicon recess or not. Vop is the operating voltage at which other key performance parameters are measured. The proposed device in this work is fabricated in an 8-inch Si photonics pilot line. The lateral SAM design not only needs the least number of layers for ion implantation but also eliminates the complexity of silicon epitaxy and the charge layer. The proposed device can achieve competitive performance at low voltage with a simpler structure. However, it also suffers from a high dark current that can be suppressed by improving the epitaxy quality. Furthermore, a lower operating voltage and higher bandwidth can be achieved by shrinking the multiplication region.

## 5. Conclusions

A low-voltage and high-speed waveguide-integrated SAM Ge/Si APD with lateral multiplication region was investigated in this work. The APD has been explored in an 8-inch CMOS pilot line without the complexity of charge layer and Si epitaxy. The device exhibits a low-level dark current and a breakdown voltage of 12 V and has a high responsivity of 15.1 A/W at −12 V, corresponding to the multiplication gain of 18.1. The opto-electrical bandwidth of 20.7 GHz is measured at −10.6 V. The maximum GBP of 232 GHz is extracted at 11.6 V. The low-bias and high-speed operation can enable energy-efficient optical communication and interconnect. Furthermore, the measurement of the large-signal and noise performance will be studied in the future. Optimization of the Ge epitaxy is being explored to suppress the Ge-Si interdiffusion and promote device performance.

## Figures and Tables

**Table 1:** Benchmarking table comparing the proposed waveguide-integrated lateral SAM Ge/Si APD with other state-of-the-art waveguide-integrated Ge/Si APDs.

Ref	Device Type	IMP Layers	Epi Si	Recess Ge	λ (μm)	*V*_op_ (V)	*I*_D_ (μA)	*R*_0_ (A/W)	*f*_3-dB_ (GHz)	GBP (GHz)
[5]	Vertical p(Ge)-i(Ge)-n(Si)	3	No	Yes	1.31	6.2	260	0.6	10.4	106
[12]	Lateral p-i-n on Ge	2	No	Yes	1.55	7	610	0.4	11	190
[13]	Lateral p(Si)-i(Ge)-n(Ge)	2	No	Yes	1.55	11	600	0.49	33	210
[15]	MSM	-	No	No	1.31/ 1.55	3.5	~1000	0.4/ 0.14	39.5	300
[19]	Vertical SACM	4	Yes	No	1.31	15	1	0.75	29.5	260
[20]	Vertical SACM	4	No	No	1.55	22	22	-	3.3	-
[21]	Vertical SACM	4	No	No	1.55	31	100	0.8	6.24	432
[22]	Lateral SACM	5	Yes	Yes	1.31/ 1.55	12	100	0.65/ 0.78	27	972
This work	Lateral SAM	2	No	No	1.55	10.6	31.9	0.8	20.7	217

## Supplementary Materials

The following are available online at https://www.mdpi.com/article/10.3390/mi13050649/s1.
