# Supplementary material for: High-Performance Waveguide-Integrated Ge/Si Avalanche Photodetector with Lateral Multiplication Region
Source: Micromachines (Basel). 2022 Apr 19;13(5):649. doi: 10.3390/mi13050649 (PMC9145896; doi:10.3390/mi13050649)
Supplement: Supplementary file 1 [file micromachines-13-00649-s001.zip › micromachines-1659321-supplementary.pdf]

High-performance waveguide-integrated Ge/Si avalanche photodetector with lateral multiplication region: supplementary material

This document provides supplementary information on "High-performance waveguide-integrated Ge/Si avalanche photodetector with lateral multiplication region". In this document, further analysis of the carrier transporting mechanism under different voltages, the relationship between multiplication gain and incident optical power, and the relationship between bandwidth and reverse voltages are included.

A. Analysis of the carrier transporting mechanism

It is a good idea to use energy-band diagrams to visualize the carrier transport mechanism at different biases. Such energy-band diagrams under different voltages could be obtained using TCAD (Lumerical Device with the version of 7.8.2779) simulation. The device model used in the simulation is shown in Figure S1. In the simulation, the top silicon and the Epi-Ge are assumed to be uniformly p-type doped with a concentration of 1.25×10^{15} cm^{-3} and 2×10^{16} cm^{-3}, respectively. The doping profile inside the n-type and the p-type heavy doped region is shown in Figure S2.

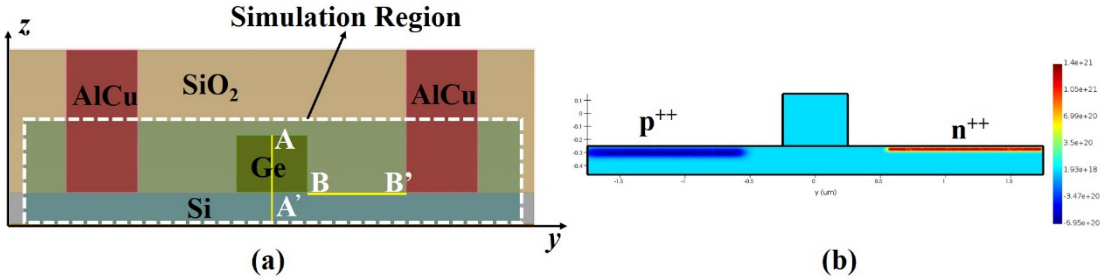

Figure S1. (**a**) Schematic of the model used in the simulation; (**b**) Doping profile of the simulation model

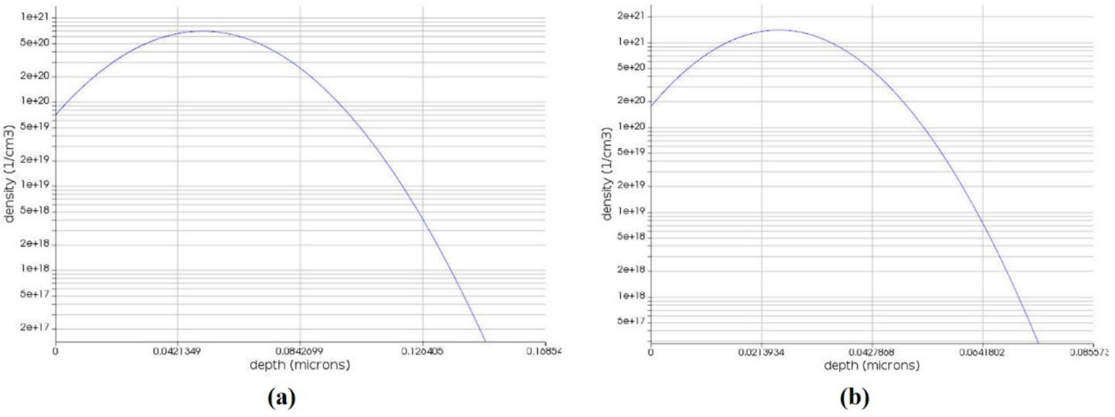

Figure S2. (**a**) Doping profile inside the p-type heavy doped region; (**b**) Doping profile inside the n-type heavy doped region

The variation of bandstructure along the A-A′ line (x= 0.0 μm, y= 0.0 μm, z= -0.47 ~ 0.15 μm) in the z-direction is recorded during the simulation at different voltages. The simulated bandst sructure is shown in Figure S3. In Figure S3, E_{c} is the conduction-band edge, E_{v} is the valence-band edge, E_{i} is the intrinsic Fermi level, E_{fn} is the electron quasi-Fermi level, and E_{fp} is the hole quasi-Fermi level. The up-row pictures are extracted from the simulation without light illumination, and the bottom-row pictures are extracted from the simulation with an incident optical power of -12.49dBm. As the hole concentration in germanium is higher than that of the underlying silicon, the holes tend to diffuse from the germanium to the underlying silicon to reach thermal equilibrium. However, the big valence-band energy offset at the Ge/Si interface hampers that diffusion. Thus, there must be holes accumulation on the side of the Ge region near the Ge/Si interface, which is why the energy band in Si is concave upward, as shown in Figure S3 (a). The built-in electric field is directed from silicon to germanium. As the reverse voltage increases from 0 V to 3 V, the direction of the electric field is reversed, and there is a hole depletion at the germanium side near the Ge/Si interface, as shown in Figure S3 (b). At the reverse voltage of 7 V, the energy band in germanium is notably concave upward, as shown in Figure S3 (c), due to the strong electric field inside the germanium region. At the same time, the majority of the germanium region should be depleted. With the reverse voltage further rise to 12 V, there is a high electric field in the germanium region and the underneath silicon region, which cause complete depletion inside these regions, as shown in Figure S3 (d).

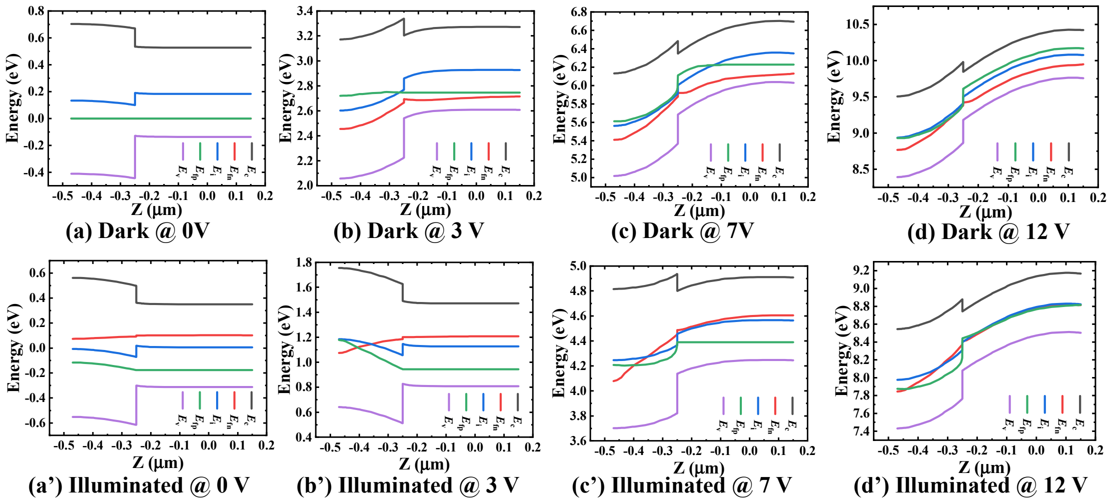

Figure S3. The simulated bandstructure along A-A′ line in the z-direction at different voltages. (**a**) Dark @ 0 V; (**b**) Dark @ 3 V; (**c**) Dark @ 7 V; (**d**) Dark @ 12 V; (**a′**) Illuminated @ 0 V; (**b′**) Illuminated @ 3 V; (**c′**) Illuminated @ 7 V; (**d′**) Illuminated @ 12 V

When the device is illuminated by light with a power of -12.49 dBm, at 0 V, the electron quasi-Fermi level, E_{fn}, is higher than the intrinsic Fermi level, E_{i}, which indicates that there are some photogenerated electron-hole pairs inside the germanium region. Due to the generated

holes accumulations at the Ge side near the Ge/Si interface, the degree of upward bending of the energy band is also higher than that in a dark environment, as shown in Figure S3 (a′). As the reverse voltage increases from 0 V to 3 V, the y-component of the electric field inside the germanium region is gradually enhanced. Photogenerated holes could be swept from the right side of the germanium to the center and pile up near the Ge/Si interface, which can be verified by the greater upward-bending of the energy band, as shown in Figure S3 (b′). The bend direction of the energy band is reversed at 5.125 V (not depicted in Figure S3 for simplicity). As the reverse voltage further rises to 7 V, there are enhanced electric field inside the germanium region, which lowers the energy barrier for electrons and facilitates the electron extraction from germanium to silicon. At the same time, the photogenerated holes can also be swept from the right side of Ge to its left side and can be extracted from the Ge to Si on the left side. Thus, the depletion of both photogenerated electrons and holes occurs at the Ge/Si interface, which can be verified by the shrinking gap among E_{fn}, E_i and E_{fp}, as shown in Figure S3 (c′). At 12 V, the Ge region should be completely depleted, the photogenerated electrons can move freely from Ge to Si and trigger the avalanche effect at the multiplication region.

It is worth noting that the above simulation and corresponding analysis are only of methodological significance. The accurate result could be obtained for practical devices by using practical parameters in the simulation, including practical interface velocity at interfaces and practical doping profile in silicon and germanium regions. However, it isn't easy to accurately measure all these parameters for the nano-scale device.

B. The relationship between multiplication gain and optical power

As can be seen in Figure 7 in the manuscript, the trend that the responsivity and multiplication gain decreases with optical power at the high voltage regime, resulting from the space charge effect. The space charge effect has been a widely topic and its impact on avalanche photodetector has been demonstrated in many literatures. At the higher optical power, a higher density of electrons is injected from the germanium region to the multiplication region, which results in an electric field collapse in the multiplication region. The electric field profile at the B-B′ line, as depicted in Figure S1 (a), is shown in Figure S4. It can be clearly seen that at the high voltage regime (notable multiplication gain), the higher the incident optical power, the lower the electric field in the multiplication region. The higher electric field leads to stronger multiplication because of the close relationship between the ionization coefficient and the electric field.

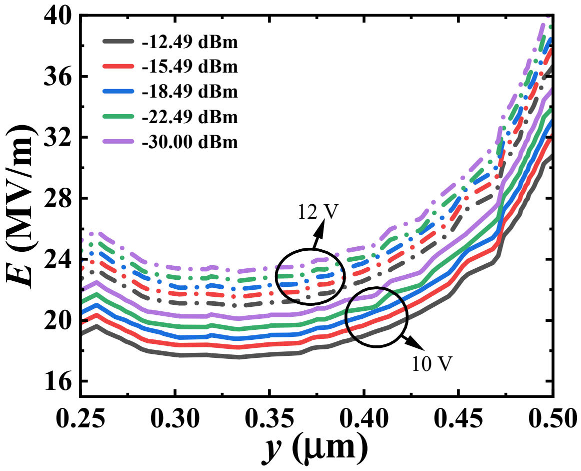

Figure S4. The electric field profile inside the multiplication region at 10 V and 12 V under different optical power.

C. The relationship between bandwidth and reverse voltage

There are four factors determining the bandwidth of an avalanche photodiode, including carrier diffusion time, carrier drift time, the RC time, and avalanche build time. The carrier diffusion time can be estimated using the following equation

$$\tau_{\mathrm{diff}} = \frac{L^2}{2D} \quad \text{(Eq-S1)}$$

where the L is the carrier diffusion length and the D is the carrier diffusion coefficient. The carrier drift time can be calculated by the following formula

$$\tau_{\mathrm{drift}} = \frac{W_{\mathrm{d}}}{v} \quad \text{(Eq-S2)}$$

where the W_d is the depletion region width and the v is the carrier drift velocity. The avalanche buildup time can be estimated using the following formula

$$\tau_{\mathrm{build}} = N(k)Mk\frac{W_{\mathrm{av}}}{v_{\mathrm{av}}} \quad \text{(Eq-S3)}$$

where the k is the ratio of the ionization coefficient of the hole to that of the electron, the M is the avalanche multiplication gain, W_{av} is the multiplication region width, v_{av} is the carrier velocity when passing through the multiplication region, which equals the saturated velocity at the high electric field, $N(k)$ is a parameter that depends on the k.

Thus the opto-electrical bandwidth of the avalanche photodiode can be expressed as

$$f_{3\mathrm{dB}} = \frac{1}{2\pi}\left(\frac{1}{\tau_{\mathrm{diff}}^2} + \frac{1}{\tau_{\mathrm{drift}}^2} + \frac{1}{\tau_{\mathrm{build}}^2} + \frac{1}{\tau_{\mathrm{RC}}^2}\right)^{-\frac{1}{2}} \quad \text{(Eq-S4)}$$

Generally, the majority of the photon-absorbing region can be depleted when the APD operates in linear mode. Thus, the carrier diffusion time has a minor effect on bandwidth. And the (Eq-S4) can be reduced to

$$f_{3\mathrm{dB}} = \frac{1}{2\pi}\left(\frac{1}{\tau_{\mathrm{drift}}^2} + \frac{1}{\tau_{\mathrm{build}}^2} + \frac{1}{\tau_{\mathrm{RC}}^2}\right)^{-\frac{1}{2}} \quad \text{(Eq-S5)}$$

As shown in Figure 9(a) in the manuscript, below 10.6 V, the bandwidth increase with the

reverse voltage, which indicates that the carrier drift time dominates the bandwidth. Because the electric field inside the germanium region increases with the bias, the carrier time used to transport the carrier should be shortened. Finally, the carrier velocity could reach its saturated velocity at some reverse voltage which is 10.6 V for our device. Consequently, the bandwidth can reach its maximum. As the reverse voltage further rises, the avalanche multiplication should be enhanced. The avalanche buildup time increases with the multiplication gain, as shown in (Eq-S3). Thus, the avalanche buildup time dominates the device bandwidth, and the bandwidth decreases with the reverse voltages.
